# Anesthesia in Dentistry

**Published:** 1914-07-15

**Authors:** B. M. Adams


### ANESTHESIA IN DENTISTRY.

BY B. M. ADAMS, D.D.S.

Read before the Jackson, Mich. Dental Society, May 6, 1914.

Anesthesia is the greatest boon that was ever discovered to alleviate suffering and permit operations to be performed that otherwise could not be endured.

Priestly discovered nitrous oxide gas in 1775.

Sir Humphrey Davy recognized its pain relieving qualities in 1800 but the credit is due Dr. Wells, a dentist of Hatrford Conn., for the discovery of its surgical properties in 1844.

He was present at a popular lecture on chemistry, given by Dr. Colton. The subject at this particular occasion was nitrous oxide or "laughing gas" and members of the audience were invited to participate. One of the participants, Mr. Cooley, inhaled the gas and gave a lively exhibition by running, jumping and falling, receiving injuries that he was unconscious of while under its influence. Dr. Wells, too, took the gas and found the sensation pleasant. These experiences suggested to him, that dental operations might be performed while the patient was under the influence of the gas, thus relieving the patient of much suffering. With this in mind he invited Dr. Colton to his office the following day and proved his conclusion by taking the gas and having Dr. Riggs, of pyorrhea fame, extract an upper third molar for him. Upon regaining consciousness he said, "I did not feel so much as the prick of a pin—a new era in tooth pulling."

In 1846 Dr. Morton discovered the anesthetic properties of ether, and Dr. Oliver Wendell Holmes, suggested the name "anesthesia" to be applied to the state produced.

In 1847 chloroform was used successfully as an anesthetic by Sir James Simpson. As soon as the news reached America Dr. Hewitt imported a small quantity from London. When it arrived he was suffering from an abscessed molar. He took a few inhalations, then lanced his gum and evacuated the pus. He then applied forceps and extracted his own tooth without discomfort. For a number of years after this he performed surgical operations upon his patients in a stage of analgesia.

Analgesia is the first stage of anesthesia, and is the condition produced whereby the patient is insensible to pain without the loss of consciousness. Complete anesthesia is accompanied with the loss of consciousness and if carried into the fourth stage the traveler doesn't return. God forbid that any Jackson dentist ever be so unfortunate as to carry his patient into the fourth stage.

Experiments were later tried upon animals by mixing nitrous oxide and oxygen. It was found that the anesthesia could be prolonged. A mixture of nitrous oxide and oxygen can be inhaled indefinitely, but this is not true of nitrous oxide alone. Nitrous oxide does not support animal or vegetable life. It is not safe to administer it even as long as one minute if all air be excluded. In the case of man the average inhalation period is fifty-six seconds. At the end of that time, fresh oxygen must be admitted or permanent asphyxia will result. (Hewitt.) Not long ago Dr. Teter of Cleveland succeeded in keeping a patient anesthetized for nearly three hours with nitrous oxide and oxygen. Dr. Andrews of Chicago was the first to use this mixture as an anesthetic. Dr. Hillischer of Vienna was the first dentist to systematically employ nitrous oxide and oxygen in definite proportions.

Ethyl Chloride was first used as an anesthetic by Heyfelder in 1848. In 1880, a committee from the British Medical Association, after experimenting upon animals made an adverse report. Its use was abandoned. But in 1895 it was revived by Carson & Theising and used to some extent by dentists.

In 1895 Dr. G. Rolland, of Bordeaux, France, was assigned the chair of anesthesia in the Bordeaux Dental School. Not being satisfied with the anesthetics in general use for dental purposes, he experimented with various mixtures until in 1899 he made public the results of his research, and called his ideal mixture "Somnoform," which is composed of Ethyl Chloride 83 percent, Methyl Chloride 16 percent, and Ethyl Bromide 1 percent, under his new formula.

Hypnotism is the ideal anesthetic, but the dental profession is not ready for it. If a person can be hypnotized and stay in any position desired and sleep for any required length of time, or as long as the hypnotist desires, and the body be made rigid and suspended between two chairs with the head resting on one and the feet on the other, and in this position hold up three or four heavy men, a weight they couldn't think of holding up if they were acting normally under their own influence, then why couldn't dental operations be performed while under this influence? They could. About twenty years ago Dr. Charles Davis, of Chicago, surgeon in charge of Temperance Hospital, hypnotized a patient for Dr. DeFord. While in this hypnotic state, Dr. DeFord and Dr. Hodgin prepared and filled a cavity with gold in an upper left central incisor. The patient did not know that he was to have a tooth filled. The tooth was a difficult one over which to adjust the rubber dam, so they had Dr. Davis make the suggestion, "dry mouth," and there was no moisture present. Cotton rolls were used to hold the lip up out of the way. Also, he was told to hold his mouth open and this he did throughout the whole operation. Dr. Davis was in an adjoining room and did not return until they had the filling completed. Then he aroused the patient, who was very much surprised when the filling was shown to him. Wouldn't it be a great relief today to both patient and operator, if every dentist could hypnotize his patients whenever they had a hypersensitive cervical cavity, a pulp removal or an extraction? Since we cannot hypnotize, thanks be to the discovery of anesthetics—we have a practical substitute we can give to deaden the pain.

Another anesthetic that patients can practice for themselves for the relief of pain, is Christian Science. Dr. DeFord relates a case of his where a lady had very sensitive teeth. All he could accomplish was to secure dryness of cavity sufficient to retain cement for a while. No attempt was made at cavity preparation. The Doctor moved to a larger place and a few years later the patient moved to the same place and hunted up her former dentist, who declined at first, to work for her on account of the nervous strain upon him when working upon such nervous cases, and of not being able to operate properly. She refused to be referred to some one else and said it would not hurt her now, that she was a Christian Scientist. So he gave her an appointment. She had cervical cavities in her anterior teeth which he thought would be a good test. He adjusted the dam, forced the gum back out of the way, made his preparations and filled them with gold and she never so much as wrinkled her forehead. He also extracted some roots for her with the same gratifying results. A very small percentage of our people needing dental services, however, have this control over their sensory nervous system. So it is up to the dentist to do painless dentistry.

If every dentist could operate without pain, the percentage of people taking care of their teeth would soon increase from 8 percent or 10 percent to a respectable proportion of the people.

Don't mistake me, I don't wish to advocate the use of an anesthetic for every patient that comes in. But that case where a dry cavity, a sharp bur and hot phenol will not suffice, where the pain, real or imaginary, seems almost unendurable, then a few whiffs of an anesthetic is advantageous in more ways than one.

When should an anesthetic be used? In all painful conditions that the dentist is called upon to treat, such as, sensitive cavity preparation, pulp removal, sometimes after an arsenical application has been made, shaping teeth for crowns or abutments whether alive or devitalized, adjusting cervical or painful clamps, treating pyorrhea, opening into teeth affected with pericementitis or acute alveolar abscesses lancing abscesses, pulpitis, opening into pulps for the purpose of making an arsenical treatment, or a cavity nearly prepared but you need more retention and your patient has stood all the nerves will allow, the nerve control is exhausted. For these operations it is seldom necessary for the patient to lose consciousness. Use the analgesia stage. With the modern appliances for nitrous oxide and oxygen, or somnoform, the patient can be kept in this condition indefinitely, and they will be resuscitated in two minutes after discontinuing the anesthetic.

May I mention a few specific cases. A patient presents with a case of acute pericementitis arising from a dead pulp. The tooth is so sore that the pressure of the tongue against it causes excruciating pain. The pulp chamber should be entered for drainage. Pressure from the excavator or bur cannot be endured without intense suffering. Here a few inhalations of an anesthetic will permit you to get drainage quickly and painlessly. In another case a child has been awake all night crying because of a case of acute pulpitis. He comes to the office all worn out from loss of sleep and hours of suffering, to which is added the dread of being hurt. The dentine must be removed and the pulp exposed. Without something to alleviate the suffering, the dentist dreads doing the work almost as much as the patient dreads having it done. Now a little gas will permit you to make the exposure, relieve the congestion and put in your emolient treatment. Another case, a patient has a labio-cervical cavity in an upper cuspid, so sensitive that you cannot make the necessary excavation and retentive form for the filling, without the patient squirming and twisting and wringing his hands, suffering intense agony, which he would *not* do, except that he might save his tooth. Here you may desensitize with a spray of ethyl chloride, or give them a little gas, go ahead and make your ideal cavity form, put in your filling and do your patient a much more satisfactory piece of work than you could hope to do without the anesthetic.

There are some cases in which only anesthesia should be used, such as extractions of various kinds from a few roots to several teeth, impacted third molars, unerupted and impacted teeth of any kind, amputation of roots where apex is diseased, curettment of necrotic bone, and various tumor formations in the mouth.

Some people are demanding anesthetics. If they have a few teeth or some roots to be extracted, they will go to a dentist that gives something so it won't hurt, or even to a "painless advertiser." You cannot blame them for not wanting to be hurt when they can have it done without pain. If you will pardon a personal reference, I had a patient that had an upper right first molar all broken down with only the roots left and they partially covered with gum tissue. She would not permit its removal with a local anesthetic and requested a general. Arrangements were made with her family physician and we went to her home and made the extraction under anesthesia. She felt perfectly satisfied because it didn't hurt her.

This brings up the idea as to what general anesthetics should be given in a dental office and what not. At the present time we have two or three that can be used successfully in the office, viz.: Nitrous Oxide and Oxygen, Somnoform and Ethyl Chloride. Ether and Chloroform should not be administered in a dental office, the patient should be taken to the hospital or his home, and a physician, preferably a specialist, should take full charge and carry the responsibility of the anesthetization.

No matter what anesthetic is given the dental surgeon should be thoroughly trained in all its physiological actions and versed in all known restoratives and resuscitation methods.

It is a very serious matter, indeed, when a patient trusts you, or puts his life in your care absolutely and becomes utterly helpless in himself and you take him down to the very border line of life and death. It is very fortunate for us that our anesthetics act upon the body in the order that they do, first, in loss of consciousness, second, loss of muscular activity, third, loss of respiration and lastly, loss of heart action. If they acted upon the circulation and respiration first, our anesthesia would be a very different problem.

Here is one place that our college curriculum could be and ought to be changed and advanced by putting in a chair of anesthesia under the supervision of a skilled anesthetist, and every student should be required to become efficient with anesthetics before he graduates.

There are a few elementary principles that are essentially necessary for success with anesthesia. First, is the preparation of the patient. No tight clothing is allowable, all bands and collars must be loosened. If the patient is a lady, the corset should be removed, and this means that a preparatory or dressing room is necessary, which can also be used for a rest room. If the appointment is made ahead the lady can come dressed ready for the operation. Dr. McClanahan, of Iowa Falls, relating one of his experiences, said: "that a lady patient insisted in reply to his request that her corset was very loose and he took her word for it. This patient as he administered the anesthetic breathed imperfectly and then ceased to breathe. Her loose corset was so tight that the doctor could not force it together to unhook it and was compelled to cut the string with a knife, before the patient could breathe again. Your lady assistant should take charge of and see that the patient is properly dressed and prepared for the anesthetic.

The stomach should be empty. This should be arranged for previously by instructions to the patient or by having the appointment two or three hours after a light meal. If the bladder has not been recently emptied, the assistant should ascertain this fact before the patient takes the chair.

If the patient is slightly nervous, have the assistant keep up a run of small talk, as this will keep the patient from dwelling upon and dreading the operation. Keep up the conversation, in a quiet and natural manner, and don't give the patient an opportunity to tell how scared he is, he will become more frightened in telling it. Mention a number of pleasing cases and laugh with them. In this way preparing the patient mentally as well as physically.

When ready for the operation adjust the apparatus and have no talking in the room while the patient is taking the anesthetic and is under its influence, except your conversation with the patient which should be suggestive of going to sleep, and of no pain, etc., In the early stages of anesthesia the hearing seems to be augmented. Even small sounds seem larger than they are. It is particularly essential that you be careful what you say. A short time ago one of our fellow dentists was having a little difficulty in getting his lady patient sufficiently under the anesthetic for the extraction. Thinking her unconscious, he swore a little, and when she recovered, she told him that he swore and also told him what he said. The anesthetic room should be the most quiet room in your office.

You should have a first class lady assistant, some of her duties I have mentioned in the preparation of the patient. She should also assist you during the operation, in watching the patient for danger signals, in attending the apparatus you are using by turning on more nitrous oxide or more oxygen, or less, as you may direct. Keep the cheek and tongue out of the way, be ready with cotton rolls or other necessary instruments All these things will materially aid in making your operation a success.

An anesthetic should never be administered to a woman in a dental office without the presence of another woman. Even the presence of a lady has a quieting effect upon the patient. No woman wants to pass into unconsciousness alone in the presence of a man with her clothing unloosened. Especially should there be a lady present if you are going to give nitrous oxide, because patients sometimes have amorous sensations and a woman might dream that improper liberties were taken by the operator. There is a case on record where the presence of mother and sister failed to convince a girl that something improper had not occurred.

The story is told on one of our Ann Arbor dentists that a colored woman had some teeth extracted under nitrous oxide anesthesia, in the presence of her husband. When she recovered she accused the dentist of improper conduct. If a person forms an opinion it is hard to change it. They say "Convince a woman against her will, she'll be of the same opinion still."

Always insert the mouth prop before the patient becomes unconscious, because if they should swallow their tongue or become nauseated before the relaxation of their muscles, respiration might stop and you could not get at the cause to remove it and death might be the result.

In prolonged operations, the gases should be heated because the rapid evaporation lowers the temperature and breathing the gas too cold and too long might cause a pneumonia or bronchitis.

What are the advantages of anesthesia and analgesia?

Some of them are, First, It enables the operator to do more thoroughly the operation to be performed.

Second, It is a less nervous strain upon the patient, perhaps because it avoids fatigue, or shock, and eliminates that exhausted feeling or collapse that follows a high nervous strain. When your patient tells you that after she was in your office for the last operation, that she had to go to bed for half a day or a day, this was bordering on shock. What can you do? Sympathise with them or laugh it off, then with sharp burs and hot phenol repeat the experience.

Third, It is a less nervous strain upon the operator, because it is much easier to work when the patient is calm and not nervous.

Fourth, It is a time saver, in a few minutes of permitted preparation, you could accomplish what it might take you many times the required time to prepare in waiting for the patient to get ready to let you work again, and then perhaps not complete the operation at that sitting.

Fifth, Prevents pain and avoids shock.

Sixth, Enables the operator to accomplish an increased amount of work in a day, thereby augmenting the receipts of his practice.

Seventh, Dignifies dentistry, elevating it to the plane of surgery.

In conclusion, let us all become expert anesthetists, and whenever analgesia or anesthesia is indicated, practice it upon our patients. Let us change our painful and dreaded operations into painless and desired dentistry. Let us elevate our profession to the level to which it belongs. Let us become the greatest benefactors of humanity.

